# Proximal policy optimization integrated with Bayesian optimization for stand structure optimization in *Pinus yunnanensis* secondary forests

**DOI:** 10.3389/fpls.2026.1840683

**Published:** 2026-06-26

**Authors:** Jianming Wang, Chenyang Lv, Jiting Yin, Yuling Chen, Shuangqing Lv, Baoguo Wu

**Affiliations:** 1 School of Mathematics and Computer Science, Dali University, Dali, Yunnan, China; 2 Dali Forestry and Grassland Science Research Institute, Dali, Yunnan, China; 3 Institute of Remote Sensing and Geographic Information System, School of Earth and Space Sciences, Peking University, Beijing, China; 4 Faculty of Surveying and Information Engineering, West Yunnan University of Applied Sciences, Dali, Yunnan, China; 5 School of Information Science and Technology, Beijing Forestry University, Beijing, China

**Keywords:** deep reinforcement learning, stand structure optimization, site factors, Bayesian Optimization, Proximal Policy Optimization

## Abstract

**Introduction:**

Secondary forests are often characterized by irrational stand structures and spatial imbalances, which constrain the full realization of their ecological functions. Existing deep learning approaches for stand structure optimization still face several limitations, including low computational efficiency, Q-value overestimation, and insufficient consideration of site-specific factors in felling constraints.

**Methods:**

To address these issues, this study focused on secondary *Pinus yunnanensis* forests on Cangshan Mountain in Dali. Based on field survey data from typical plots, a stand structure optimization model was developed with individual trees as decision-making units. At the algorithmic level, the proximal policy optimization (PPO) algorithm was introduced to replace the Deep Q-Network (DQN), employing proximal clipping to reduce Q-value overestimation and stabilize high variance. This approach was further integrated with Bayesian optimization (BO) for automatic hyperparameter tuning, thereby establishing a PPO-BO framework. In terms of regulatory constraints, slope was incorporated as a key site factor, with trees located on slopes steeper than 45° excluded from felling to ensure ecological suitability. Additionally, six optimization schemes were designed, comprising three tree selection strategies combined with a multi-objective optimization framework, and their performance was systematically evaluated.

**Results:**

The results demonstrated that the PPO-BO algorithm consistently achieved 19 higher maximum objective function values across five circular plots (0.4475, 0.5075, 0.5146, 0.5133, and 0.5255) compared with the DQN algorithm (0.4421, 0.4973, 0.5051, 0.4947, and 0.5057), while also reducing overall training costs. Following selective felling, the spatial structure indices of each plot improved markedly, with the overall stand structure gradually approaching that of natural forests.

**Discussion:**

Overall, this study provides new insights into the optimization of stand structure in secondary forests and enriches the theoretical and methodological framework for sustainable forest management.

## 1 Introduction

Secondary forests are plant communities that naturally regenerate following the destruction of primary forests, and are also referred to as regenerated forests. After human-induced disturbances or extreme natural events, the structure of forest stands undergoes profound changes. In China, forest communities are now predominantly composed of natural forests that have either remained undisturbed or have recovered through natural regeneration (Gangying et al., 2009). The eastern slope of Cangshan Mountain, situated in the Dianxi region of Dali Bai Autonomous Prefecture in northwestern Yunnan Province, features foothills with a subtropical climate. Local forest resources are mainly composed of *Pinus yunnanensis* and artificially regenerated secondary forests. While many of these stands retain their natural attributes, only limited areas have been affected by human modification or production activities. However, the majority are middle-aged or young stands, characterized by simple composition, unreasonable structure, and widespread problems such as quality degradation, frequent fires, and pest or disease outbreaks (Yuan, 2010; Xuan et al., 2024). Forest stand structure results from long-term ecological succession and the combined influence of environmental factors. Quantifying structural characteristics is a prerequisite for forest structural management (Guoyu and Ruide, 2003) and holds significant theoretical and practical implications for the sustainable management of secondary forests (Lingbo et al., 2013; Jing et al., 2023). Stand spatial structure specifically refers to the distribution and arrangement of trees, including species composition, tree size, and spatial pattern. Optimising stand structure enhances long-term productivity and economic benefits, thereby supporting sustainable forest development (Huang et al., 2019; Ali, 2019; Tang et al., 2020; Von Gadow and Hui, 2002). This optimization problem is inherently a dynamic multi-objective optimization task. Algorithms such as genetic algorithms (Dongsheng et al., 2022), particle swarm optimization (PSO) (Jianjun et al., 2013), and simulated annealing (Kang et al., 2020) have been widely applied to such problems, as they adapt to environmental dynamics and balance multiple objectives. Nevertheless, these methods are limited by high computational complexity, slow convergence, and unstable accuracy, limits their dynamic adaptability and long-term optimization performance. Consequently, there is an urgent need for approaches capable of adaptively handling complex spatial states, achieving long-term optimization, and supporting high-dimensional decision-making. Reinforcement learning offers such potential, given its strengths in decision optimization and adaptability.

Reinforcement learning (RL) operates by guiding intelligent agents through a reward-punishment mechanism. Agents interact with the environment, receive feedback, and iteratively adjust their policies to maximise cumulative rewards. This framework effectively addresses the shortcomings of traditional algorithms when tackling complex, dynamic environments (Waubert de Puiseau et al., 2024). RL has already shown promise in multi-objective optimization of forest stand structure. For example, Xuan et al. (2023) modelled tree selection as the action space of an RL agent and applied the Q-learning algorithm to solve this problem. To further evaluate the effectiveness of RL methods, that study compared different tree selection strategies: PSO was employed to generate heuristic harvesting plans (Qiu et al., 2023), while Monte Carlo simulation was used for random decision evaluation (Boston and Bettinger, 1999). Multi-indicator analyses confirmed the feasibility and superior performance of Q-learning in stand structure optimization. However, classical RL algorithms encounter difficulties when dealing with large, continuous state spaces and complex action spaces, such as those in secondary *Pinus yunnanensis* forests. Challenges include the curse of dimensionality and limitations in feature representation. Deep reinforcement learning (DRL) addresses these challenges by combining the representational power of deep learning with the optimization capacity of RL. Zhao et al. (2024) applied the deep Q-network (DQN) algorithm to represent tree selection decisions as agent actions, integrating various tree removal strategies into a DRL framework. This study validated the feasibility and effectiveness of DQN for stand structure optimization. DRL demonstrates remarkable computational efficiency, stability, and generalisation when handling high-dimensional states and complex optimization tasks (Li et al., 2020; Waschneck et al., 2018; Oroojlooyjadid et al., 2022). Yet, issues remain, including susceptibility to local optima and low exploration efficiency during early training. Hyperparameter optimization can significantly improve DRL performance by guiding exploration strategies, reducing randomness, and enhancing solution efficiency. Bayesian optimization (BO) provides a principled method for hyperparameter tuning in machine learning and deep learning. By modelling the nonlinear relationship between parameters and performance through Gaussian processes, and balancing exploration and exploitation with acquisition functions, BO enhances adaptability, convergence speed, and robustness in complex tasks such as forest stand optimization. For instance, Ishikawa et al. (2025) demonstrated the effectiveness of BO in selecting optimal machine learning models and hyperparameters, while Onorato (2024) applied BO to neural network tuning, achieving effective balance between exploration and exploitation.The proximal policy optimization (PPO) algorithm further strengthens this framework. PPO supports both discrete and continuous action spaces, stabilises training through policy clipping, and promotes adaptive exploration via policy entropy. These features make it particularly well-suited for high-dimensional state spaces and sparse reward settings. Schulman et al. (2017) demonstrated PPO’s advantages over DQN in continuous and high-dimensional action spaces, where PPO avoids Q-value overestimation by directly optimising the policy through policy gradients. BO has also been applied to enhance various DRL algorithms, with studies such as Victoria and Maragatham (2021) showing that Bayesian hyperparameter optimization improves model performance, reduces training costs, and enhances robustness. Despite its success in other domains, PPO has not yet been applied to forest stand optimization. Stand structure optimization involves multiple interacting indicators, often nonlinear, non-convex, and conflicting, alongside spatial adjacency and balance constraints. These conditions generate extremely large search spaces prone to local optima. PPO is particularly suitable here, as it can repeatedly interact with the environment, leveraging reward signals to adaptively optimise harvesting strategies. Unlike heuristic or purely random search methods, PPO requires fewer trials to find effective solutions, an important advantage given the computational cost of stand structure metric calculations. This highlights PPO’s potential as a powerful tool for multi-objective optimization in secondary forest management.

The primary regulatory measures for optimising stand structure are selective felling and replanting. Since tree growth is inherently constrained by site conditions and spatial competition, the appropriate selection of felling constraints and replanting locations becomes critically important. In previous felling optimization experiments, Zhao et al. (2024) did not incorporate the influence of topographic factors into the optimization process. However, steep slopes often serve as critical catchment and water-retention zones, playing an indispensable role in soil and water conservation. Therefore, determining a scientifically justified threshold slope gradient beyond which felling operations should be restricted (or below which they should be prohibited) represents an important and practically meaningful research question.

In summary, the integration of deep reinforcement learning with Bayesian optimization enables automatic hyperparameter tuning, thereby reducing manual tuning effort and time while identifying optimal hyperparameter combinations for specific forest stand environments. Accordingly, this study applied the PPO-BO algorithm to optimise the structure of secondary *Pinus yunnanensis* forests and evaluated its effectiveness through simulated thinning experiments. Three different tree selection strategies were employed, with the DQN algorithm introduced as a benchmark for comparison. Through these simulations, the study systematically evaluated the performance and applicability of the PPO-BO framework in stand structure optimization. The findings provide new technical insights and decision-support strategies for the intelligent management and structural optimization of secondary *Pinus yunnanensis* forests.

## 2 Materials and methods

### 2.1 Overview of the study area

**Figure 1:**
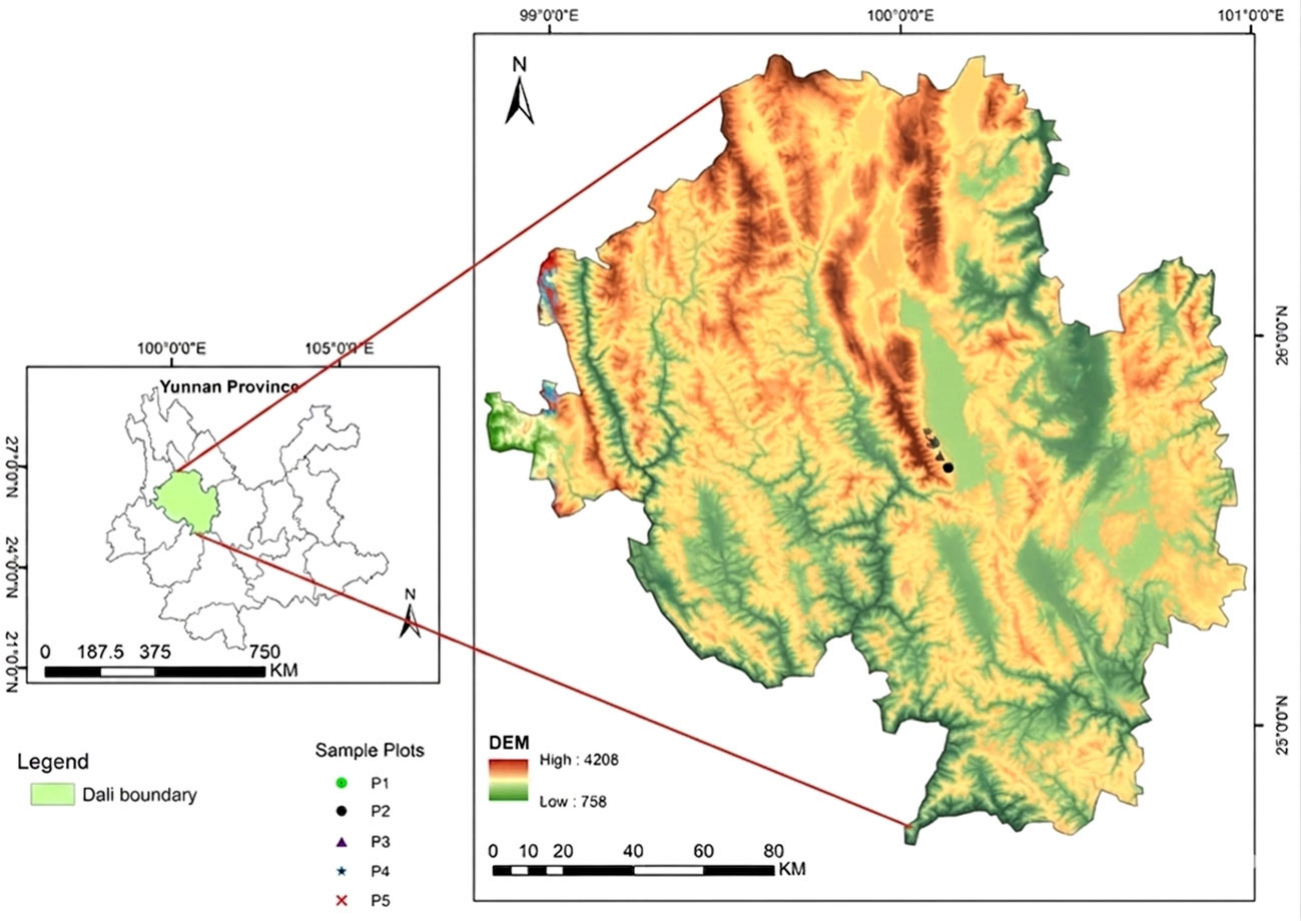
Study area.

The study area is located in the Cangshan Mountain Range (25°34^′^-26°00^′^*N*, 100°55^′^-100°11^′^*E*) within the Dali Bai Autonomous Prefecture, Yunnan Province, China. It covers approximately 293 *km*^2^ and ranges in elevation from 1,966 to 4,122 meters above sea level. The region features a typical subtropical plateau monsoon climate, characterized by an average annual temperature of 15.5°C (Zhang et al., 2022), abundant sunshine, and a highly uneven seasonal distribution of precipitation-approximately 83% of the annual rainfall occurs between May and October. The dominant soil type is red soil. Vegetation in the area is primarily composed of *Pinus yunnanensis* as the principal species, with associated species including Huashan pine (*Pinus armandii*), Chinese cork oak (*Quercus acutissima*), and Southwestern birch (*Betula alnoides*). The shrub layer consists of species such as *Vaccinium bracteatum*, *Rhododendron microphyton*, and other subtropical plants including *Gaultheria griffithiana* and *Ternstroemia gymnanthera*. A map of the study area is presented in **Figure 1**.

### 2.2 Data collection and processing

In the autumn of 2024, field surveys were conducted in selected sample plots within the study area.

A total station (GTS-2002) was used to accurately map five circular sample plots. Key geographic and topographic data-including geographic coordinates, elevation, slope gradient, slope aspect, and plot radius- were carefully recorded for each plot (Packalen et al., 2023). According to the criterion of DBH ≥ 5 cm, all living trees within each plot were surveyed. Tree-level data collected included species name, relative spatial coordinates, diameter at breast height (DBH), tree height, crown width, and other essential growth metrics. The relative coordinates of individual trees were measured using the total station with millimeter-level precision. A summary of the basic structural characteristics of the sample plots is provided in **Table 1**.

**Table 1:** Basic information about the site.

Plot	Altitude/m	Slope/^·^	Slope position	Slope aspect	Average diameter at breast height/cm	Average tree height/m	Plot radius/m	Stand density (*trees* · *hm*−2)	Tree species composition
P1	2254	13.45	middle	East Slope	17.10	11.97	35	1603	8 *Pinus yunnanensis* + 2 *Pinus armandii - Betula albosinensis* *- Rhododendron microphyton - Ternstroemia gymnanthera*
P2	2273	16.16	middle	South Slope	13.79	9.39	32	2182	7 *Pinus yunnanensis* + 3 *Pinus armandii*
P3	2205	17.70	middle	North-East Slope	14.50	9.30	20	2109	7 *Pinus yunnanensis* + 3 *Pinus armandii + Betula alnoides* *- Quercus acutissima - Vaccinium bracteatum - Gaultheria griffithiana*
P4	2138	5.10	middle	North-East Slope	14.26	10.94	19	2618	10 *Pinus yunnanensis - Quercus mongolica*
P5	2253	15.25	middle	South-East Slope	16.03	9.57	30	2631	8 *Pinus yunnanensis* + 1 *Pinus armandii* + *Quercus acutissima* - *Vaccinium bracteatum* - *Betula alnoides* - *Camellia sinensis*

### 2.3 Determination of spatial structural units and edge correction

In this study, the Voronoi diagram method was used to construct the spatial structural unit of the forest stand, which can reflect the spatial structural characteristics of the forest stand more comprehensively and reasonably by considering the specific spatial relationship of each centre tree (Liu et al., 2023). The specific implementation process is as follows: firstly, based on the measured coordinates of forest trees, R 4.2.0 software was used to generate a Voronoi diagram, in which each polygon represented a spatial structure unit composed of the centre tree and its adjacent trees. In order to eliminate the influence of the sample plot boundary effects on the calculation of spatial structure parameters, this study adopted the distance buffer method for boundary processing: a circular buffer was set up within 2 m inward from the sample plot boundary. When calculating the spatial structure index, the trees in the buffer zone only participated in the construction of the spatial structure unit as neighbouring trees, and were not analysed as the central trees. This method effectively avoided the parameter calculation error caused by the boundary effect.

### 2.4 Stand structure index

Quantifying stand structure is a prerequisite for its effective optimization. In this study, both non-spatial and spatial indicators were used to describe forest stand structure. Non-spatial indicators included the number of DBH classes, number of tree species, and canopy closure. Spatial structural indicators included the angular scale, species mingling degree, canopy competition index, stratification index, and openness ratio. Specifically: The angular scale reflects the horizontal distribution pattern of trees; The mingling degree indicates the degree of species mixing or isolation; The canopy competition index quantifies the intensity of inter-tree competition; The stratification index describes the vertical layering pattern of tree crowns; The openness ratio assesses the level of light availability in the understory.

#### 2.4.1 Non-spatial structural indicators

1. Tree diameter classes

In this study, a diameter at breast height (DBH) of 6 cm was used as the lower threshold, and the trees were categorized into diameter classes using 2 cm intervals. The results indicated that the structural complexity of the stand was positively correlated with its growth quality-i.e., the finer the classification, the better the overall growth condition of the stand. During the optimization process, it is essential to preserve the diversity of diameter classes, ensuring that the number of classes does not decrease after harvesting, as shown in Equation 1:

(1) $$D=D_{0}$$

Where *D*_0_ is the number of diameter classes of trees in the stand before harvesting and *D* is the number of diameter classes of trees in the stand after harvesting.

2. Number of tree species.

Selective logging must ensure the preservation of species composition within the stand to prevent local extinction of certain species caused by human disturbance. The species richness should remain stable before and after the operation, as expressed in Equation 2:

(2) $$T=T_{0}$$

Where *T*_0_ is the number of tree species in the stand before harvesting and *T* is the number of tree species in the stand after harvesting.

3. Intensity of harvesting

Harvesting intensity has a direct impact on the long-term sustainability of the forest. It is generally recommended that the annual harvest volume should not exceed the annual growth volume. For secondary forests dominated by *Pinus yunnanensis*, previous studies (Han et al., 2011; Su et al., 2010) have shown that harvesting intensity should be strictly controlled below 35% to maintain forest health, as shown in Equation 3:

(3) $$N\geq N_{0}\left(1-35\%\right)$$

Where *N*_0_ is the total number of trees in the stand before harvesting and *N* is the total number of trees in the stand after harvesting.

4. Canopy Density(*Cd*).

In forest management, maintaining continuous canopy cover is a fundamental objective. According to national forestry standards, a canopy density (Cd) of 0.7 or higher indicates that the stand has achieved continuous canopy cover, as defined in Equation 4:

(4) Cd≥0.7

#### 2.4.2 Spatial structure indicators

1. Neighborhood Comparison(*U*) (Aguirre et al., 2003)

It describes the degree of size differentiation and competition among trees within a stand, expressed as the ratio of neighboring trees with a diameter at breast height (DBH) greater than that of the target tree to the total number of neighboring trees, as defined by Equation 5:

(5) $$U_{i}=\sum_{j=1}^{n}k_{ij}$$

In the formula, *U_i_* represents the size ratio of target tree *i*, *k_ij_* is a discrete variable, when the diameter at breast height of neighbouring wood *j* is larger than the diameter at breast height of target tree *i*, *k_ij_* = 1, otherwise *k_ij_* = 0. The smaller *U_i_* is, the greater the dominance of the target tree is. The value range of *U_i_* can be divided into five class intervals: [0,0.25], (0.25,0.5], (0.5,0.75], (0.75,1], which characterises the degree of dominance of the target tree in the stand as dominant, sub-dominant, mediocre, inferior, and absolutely inferior.

2. Canopy competition index (Wang, 2017)

To quantify the intensity of competition among trees, this study adopts a canopy-based competition index. A spatial overlap model of tree canopies is constructed by integrating morphological parameters such as the projected canopy area and vertical canopy length. This model characterizes the level of resource competition experienced by individual trees during their growth processes, offering a structural perspective on inter-tree interactions. The canopy competition index is calculated using Equations 6–9:

(6) $$CI_{i}=\frac{1}{Z_{i}}\times \sum_{j=1}^{n}AO_{ij}\times \frac{L_{j}}{L_{i}}$$

In the formula, *CI_i_* denotes the canopy competition index of target tree *i*, *Z_i_* is the projected area of the canopy of target tree *i*, *L_j_*= *H_j_*× *Cw_j_*× *CL_j_*(height of competing tree *j* × crown width of competing tree *j* × crown length of competing tree *j*), *L_i_*= *H_i_*× *Cw_i_*× *CL_i_*(height of reference tree *i* × crown width of reference tree *i* × crown length of reference tree *i*), and *AO_ij_*denotes the area of overlapping canopies between target tree *i* and competing wood *j*, which is 1 if there is no overlap. The formula for calculating *AO_ij_*is as follows:

(7) $$S_{0}=\frac{Cw_{i}^{2}}{2}\sum_{j=1}^{n}\arccos\;(\frac{q_{j}^{2}}{2Cw_{i}^{2}}-1)-\frac{1}{4}\sum_{j=1}^{n}q_{j}\sqrt{4Cw_{i}^{2}-q_{j}^{2}}$$

(8) $$S_{1}=\frac{1}{2}\sum_{j=1}^{n}[Cw_{j}^{2}\arccos\;(1-\frac{4Cw_{i}^{2}-q_{j}^{2}}{2Cw_{j}^{2}})-\frac{\sqrt{4Cw_{i}^{2}-q_{j}^{2}}}{2}]\sqrt{4Cw_{i}^{2}-(4Cw_{i}^{2}-q_{j}^{2})}$$

(9) $$AO_{ij}=S_{0}+S_{1}$$

Where *S*_0_ is the total area shaded by *n* competing trees to target tree *i*, *S*_1_ is the total area shaded by target tree *i* to *n* competing trees, $q_{j}=\frac{L_{ij}^{2}-\left(Cw_{j}^{2}-Cw_{i}^{2}\right)}{L_{ij}}$ is the distance between competing wood *j* and target tree *i*, *Cw_i_* is the crown radius of target tree *i*, *Cw_j_* is the crown radius of competing wood *j*, and *n* is the number of competing wood plants.

3. Stratification Index(S)

As an important index characterising the vertical spatial distribution of forest trees, the calculation of the forest layer index (Zhou et al., 2022) is based on the product of the proportion of inter-storey differences between neighbouring and object trees and the structural heterogeneity of spatial units. However, the premise for the construction of this index assumed that the study area is an ideal horizontal terrain and failed to incorporate the influence of actual terrain factors. Due to the high topographic complexity and significant elevation differences in this study area, the applicability of the traditional forest layer index is limited. For this reason, this study developed the forest difference index under the framework of the traditional algorithm by introducing terrain correction factors (Zhao et al., 2024).

The stratification algorithm for the forest difference index is based on the vertical spatial division of the dominant height of the stand: the canopy structure is divided into three vertical gradients, in which the upper layer of the stand is defined as individuals with tree heights greater than or equal to 2/3 of the dominant height, the middle layer of the stand as individuals with tree heights within the interval of 1/3 to 2/3 of the dominant height, and the lower layer of the stand as individuals with tree heights less than or equal to 1/3 of the dominant height. In order to eliminate the interference of topographic factors, this method uses the arithmetic mean height of the 100 tallest standing trees per hectare in the sample plot as the dominant height reference value (Xiangdong et al., 2018; Sharma et al., 2002). The stratification index is expressed in Equations 10–12:

(10) $$S_{i}=\frac{z_{i}}{3}\times \frac{1}{n}\sum_{j=1}^{n}\left(1-\frac{\left|FL_{i}-FLj\right|}{max\left(\left|FL_{i}-FLj\right|,1\right)}\right)$$

(11) $$FL_{i}=\{\begin{array}{l} -1,H_{i}\leq \frac{1}{3}H_{d}\\ 0,\frac{1}{3}H_{d}<H_{i}\leq \frac{2}{3}H_{d}\\ 1,H_{i}\geq \frac{2}{3}H_{d} \end{array}$$

(12) $$H_{d}=\frac{1}{100A}\sum_{i=1}^{100A}\left(H_{sort\left(i\right)}+E_{sort\left(i\right)}\right)$$

*S_i_* denotes the forest difference index of target tree *i*, *z_i_* denotes the number of forest layers in the spatial structure unit to which target tree *i* belongs, *FL_i_* denotes the classification of target tree *i* in the vertical hierarchy, *H_i_* denotes the height of target tree *i*, *H_d_*denotes the dominant height, *A* denotes the area of the sample plot per hectare, *H_sort_*_(_*_i_*_)_ denotes the height of the *i*-th tree in the first 100*A*, and *E_sort_*_(_*_i_*_)_ denotes the relative elevation of the *i*-th tree in the first 100*A*. The closer the forest difference index is to 1, the more complex the stratification pattern of the stand is in the vertical direction.

4. Full hybridisation (Mengping et al., 2004)

In order to quantify the spatial segregation characteristics of tree species, this study used a comprehensive assessment method based on the proportional homogeneity of species composition to characterise the level of tree species diversity at the stand scale by integrating the relative multiplicity of distributional characteristics of each species, which was expressed as Equation 13:

(13) $$Mc_{i}=\frac{M_{i}}{2}[1-\frac{1}{{\left(n+1\right)}^{2}}\sum_{j=1}^{s_{i}}n_{j}^{2}+\frac{n_{i}}{n}]$$

Where *Mc_i_* is the full hybridisation degree of target tree *i*, *n_i_* is the number of different species in the adjacent wood, *n_j_* is the number of trees of species *j* in the adjacent wood, and *s_i_* is the number of species in the spatial structural unit in which target tree *i* is located. *M_i_* is the mixing degree, $M_{i}=\frac{1}{n}\sum_{j=1}^{n}v_{ij}$. When the target tree *i* and the neighbouring wood *j* are of the same species, *v_ij_* is 0, and vice versa is 1.

5. Angular scale (Zhang et al., 2018)

Is used to describe the spatial distribution pattern among forest trees and is defined as the proportion of the number of angles (the smaller angles between neighboring trees) smaller than the standard angle *α*_0_ ( $\alpha_{0}=\frac{360^{\circ }}{n+1}$) that form *n α* angles, which is expressed as Equation 14:

(14) $$W_{i}=\frac{1}{n}\sum_{j=1}^{n}z_{ij}$$

Where *W_i_* denotes the angular scale, *z_ij_* is a discrete variable, when the *j*-th *α* angle is smaller than the standard angle *α*_0_, *z_ij_* is 1, and vice versa is 0. The values of *W_i_* can fall in the five intervals of [0,0.25], (0.25,0.5], (0.5,0.75], (0.75,1], which denote absolute uniform, uniform, random, relatively aggregated and aggregated, respectively. The range of angular scale mean values of the ideal forest stand should be in the interval [0.475,0.517].

### 2.5 Construction of a forest stand structure optimization model

#### 2.5.1 Restrictive condition

To accurately quantify and optimise the spatial structure of forest stands in the study area, a mature model capable of integrating multiple structural indicators is required. Based on a comprehensive review of the literature, this study adopted the forest stand structure optimization model proposed by Zhao et al. (2024). This model systematically incorporates key structural parameters, including angular scale, size ratio, mingling degree, forest layer index, and canopy competition index. Its effectiveness has been thoroughly validated in studies on secondary *Pinus yunnanensis* forests. In the present study, we strictly followed Zhao’s model framework while introducing a deep reinforcement learning algorithm combined with Bayesian optimization to further enhance stand structure optimization.

1. Spatial structure constraints

The spatial structure constraints aim to optimise the spatial distribution pattern of the stand, and require that the spatial structure parameters of the optimised stand are not inferior to those of the pre-optimised stand, which include: decreasing the size ratio, angular scale and canopy competition index to reduce the degree of competition and size differentiation; increasing the all-mixedness degree and the stand difference index to increase the degree of species mixing and the richness of the vertical structure; and making the horizontal distribution of the stand tend to be randomly distributed, so that the spatial heterogeneity of the stand can be optimised. The 45° slope threshold adopted in this study is grounded in multiple lines of evidence from the fields of geomorphology, hydrology, and Chinese forestry regulation. First, research on landslide mechanics indicates that slopes with gradients between 10° and 45° - particularly those with a steep lower section, a gentle middle section, and a steep upper section - constitute terrain most susceptible to shallow translational landslides (He, 2025). At angles exceeding 45°, soil shear stress surpasses shear strength under saturated conditions, and the stabilising contribution of tree root networks becomes critical; felling in these zones significantly elevates the risk of mass movement. Second, studies on surface runoff and soil erosion have demonstrated that erosion intensity increases sharply once slope gradients exceed approximately 40°–45°, beyond which loss of vegetation cover produces disproportionate increases in sediment yield (Sun et al., 2021; Simelane et al., 2024). The eastern slope of Mount Cangshan, where the study plots are located, falls within such designated protected areas. Taking into account the aforementioned topographical and hydrological considerations, the 45° threshold is well-founded both scientifically and operationally, and may serve as a criterion for excluding candidate trees for felling. Furthermore, a vegetation ecology study conducted in the Cangshan area - the study region of the present research - demonstrated that when slope gradients exceed 45°, the distribution areas of nearly all major vegetation types decline significantly, indicating that such terrain is ecologically unsuitable for the survival of most forest community types (Li et al., 2025). This finding provides direct site-specific evidence that slopes steeper than 45°constitute a critical ecological threshold, beyond which vegetation survival and the ecological functions of tree root systems in maintaining soil stability are severely impaired. Accordingly, this study established 45° as the critical slope angle for felling restriction. All trees located on slopes steeper than 45° were excluded from the pool of candidate trees for felling and were included only in the assessment of stand spatial structure and the calculation of replanting configurations.

2. Non-spatial structural constraints

Non-spatial constraints are used to maintain the basic structural characteristics of the stand to ensure that the optimised stand meets the requirements for sustainable management, specifically: the number of tree species and the number of diameter classes will not be reduced to maintain the biodiversity; the canopy density will not be lower than 0.7 to ensure the stand cover; and the intensity of harvesting will be controlled to 35% or less to avoid excessive disturbance as defined in Equation 15.

(15) $$s.t.\{\begin{array}{l} \bar{Mc}\geq \bar{Mc_{0}}\\ \bar{S}\geq \bar{S_{0}}\\ \bar{U}\geq \bar{U_{0}}\\ \bar{CI}\geq \bar{CI_{0}}\\ \left|\bar{W}-0.496\right|\leq \left|\bar{W_{0}}-0.496\right|\\ D=D_{0}\\ T=T_{0}\\ Cd\geq 0.7\\ N\geq N_{0}\left(1-35\%\right)\\ Slope<45^{\circ } \end{array}$$

Where $\bar{MC}$, $\bar{S}$, $\bar{U}$, $\bar{CI}$, $\bar{W}$, *D*, *T*, *Cd*, *N* denotes the values of total mixedness, stand difference index, size ratio, canopy competition index, angular scale, number of diameter classes, number of tree species, degree of depression, and felling intensity of the optimised stand, respectively. $\bar{Mc_{0}}$, $\bar{S_{0}}$, $\bar{U_{0}}$, $\bar{CI_{0}}$, $\bar{W_{0}}$, *D*_0_, *T*_0_, *N*_0_ denotes the values of total mixedness, stand difference index, size ratio, canopy competition index, angular scale, number of diameter classes, number of tree species, and felling intensity of the pre-optimised stand, respectively, *Slope* represents the removal of slope constraints.

#### 2.5.2 Models

Optimization of stand structure is a typical multi-objective optimization problem, in which the subobjectives (e.g. spatial distribution, species mixing, competitive relationships, etc.) are both constrained and closely related to each other, and it is difficult to achieve the theoretical optimum for all objectives at the same time. Therefore, it is necessary to coordinate the trade-offs among sub-objectives through multi-objective planning methods from the perspective of overall optimization, in order to achieve the global optimal allocation of forest stand structure.

Based on this, this study selected five key spatial structure parameters, namely angular scale, size ratio, full mixing degree, stand difference index and canopy competition index, and used the multiplication and division method’ (Yuhan et al., 2018) to construct a multi-objective planning framework. The objective function of the multi-objective harvesting optimization model for stand structure was established by integrating the positive and negative (need to reduce, e.g., W, parameters). The objective function is established as Equation 16:

(16) $$maxL=\frac{1}{N}\sum_{i=1}^{N}\frac{\frac{1+Mc_{i}}{\delta_{Mc}}\cdot \frac{1+S_{i}}{\delta_{S}}}{\frac{1+U_{i}}{\delta_{U}}\cdot \frac{1+CI_{i}}{\delta_{CI}}\cdot \frac{1+\left|W_{i}-0.496\right|}{\delta_{\left|W-0.496\right|}}}$$

In the stand structure optimization model constructed in this study, *W_i_*, *Mc_i_*, *S_i_*, *U_i_* and *CI_i_* represent the angular scale of the target trees, the degree of all-mixedness, the stand difference index, the number of size ratios and the canopy competition index, respectively, and the standard deviation of each parameter is denoted as *δ_W_*, *δ_Mc_*, *δ_S_*, *δ_U_* and *δ_CI_*, while *N* is the total number of trees in the stand. The model achieves the following spatial structure improvement through multi-objective optimization: under the premise of ensuring that the quality of the existing spatial structure is not degraded, it requires a monotonically increasing trend in the full mixing degree and stand difference index, and a monotonically decreasing trend in the number of size ratios and the canopy competition index; at the same time, based on the theoretical characteristics of a randomly distributed forest stand, we take the median of the angular scale mean interval [0.475,0.517] of 0.496 as the optimization benchmark, and by minimising |*W* − 0.496| to bring the horizontal distribution of the forest stand closer to a random pattern. This comprehensive optimization strategy aims to simultaneously improve the spatial heterogeneity, species mixing and vertical structure diversity of the stand, while reducing the competitive pressure among trees, and ultimately achieving the overall optimization of the stand structure.

#### 2.5.3 Methods for determining trees to be felled

Having identified the trees to be retained following optimization, it is necessary to establish a set of objective and quantifiable criteria for selecting trees to be felled in order to implement specific logging operations. This study directly adopted the tree selection methods proposed by Zhao et al. (2024). The three methods differ fundamentally in their selection criteria, indicator dimensions, and prioritisation logic, representing a progressive framework that evolves from random selection to multi-parameter comprehensive evaluation and finally to spatial pattern-oriented optimization, as detailed below.

1. Random selection (Tang et al., 2004)

Under the constraint of a prescribed felling intensity, no structural selection criteria are imposed on the trees within the plot; instead, the set of trees to be removed is selected entirely at random. This method does not rely on any spatial structural indicators, and felling decisions are independent of both the spatial positions of trees within the competitive environment and species composition. Its primary purpose is to serve as an uninformative baseline for comparison with the subsequent structured methods. By contrasting theoptimization outcomes of the random method with those of the structured approaches, the actual benefit of incorporating spatial structural information into felling decisions can be quantitatively assessed.

2. Selection of trees to be harvested based on multi-parameter comprehensive evaluation (Yitong, 2019)

This method integrates five spatial structure parameters—scale of variation, total heterogeneity, forest diversity index, size ratio, and canopy competition index—into a unified evaluation framework. A composite index is constructed for each individual tree as shown in Equation 17, and harvesting priorities are determined through ascending ranking, with the highest-ranked individuals included in the candidate set for removal.

(17) $$L_{i}=\frac{\frac{1+Mc_{i}}{\delta Mc}\cdot \frac{1+S_{i}}{\delta_{S}}}{\frac{1+U_{i}}{\delta_{U}}\cdot \frac{1+CI_{i}}{\delta_{CI}}\cdot \frac{1+W_{i}}{\delta_{W}}}$$

In the formula, *W_i_*, *Mc_i_*, *S_i_*, *U_i_* and *CI_i_* are the angular scale, total mixing degree, forest difference index, size ratio, and canopy competition index of the object tree, respectively, and *δ_W_*, *δ_Mc_*, *δ_S_*, *δ_U_*and *δ_CI_* are the standard deviations of each structural parameter, respectively. Compared with random selection, the key distinction of this method lies in its comprehensive coverage across multiple dimensions. It simultaneously considers spatial distribution patterns, species isolation, vertical structure, competitive intensity, and size differentiation, aiming to identify individuals most in need of removal from a holistic structural perspective. However, the equal-weighted integration of multiple indicators may dilute the contribution of individual dimensions, resulting in relatively weaker specificity with respect to particular structural issues, such as spatial competition.

3. Selection of trees to be removed based on spatial pattern optimization (Zhang et al., 2019)

The theoretical range of the angular scale mean for an ideal forest stand is [0.475, 0.517], where when |*W* − 0.496| approaches 0, it indicates that the horizontal distribution pattern of the forest stand is closest to a random distribution state. Based on this, this study employs a multi-indicator collaborative screening strategy to determine trees for removal: first, select trees with angular scale values significantly deviating from the theoretical value (0.496) (i.e., those with larger |*W* − 0.496| values); Second, among these candidate trees, those with higher size ratio (reflecting competitive disadvantage) and crown competition index (indicating spatial competition intensity) are prioritised for removal. Through this dual screening mechanism, both the spatial distribution pattern of the stand is optimised and the competitive pressure between trees is effectively reduced, ultimately yielding a scientifically reasonable set of trees to be removed. This method focuses on the targeted regulation of stand-level spatial distribution patterns. It selects three indicators—angular scale, size ratio, and canopy competition index—and employs a two-stage screening mechanism to identify trees for removal. First, the deviation of the angular scale from the theoretical value of random distribution (i.e., large |*W* − 0.496|) is used as the primary screening criterion to prioritise individuals exhibiting anomalous spatial configurations. Building on this, higher values of the size ratio and canopy competition index are used as secondary criteria to identify trees experiencing strong competitive disadvantages. Compared with Method (2), the core distinction lies in its more focused objective orientation: it prioritises the correction of spatial aggregation patterns, while competition-related indicators serve a supplementary role. As a result, this method demonstrates greater specificity in improving stand-level distribution patterns, although its capacity to regulate species composition and vertical structure is relatively limited.

### 2.6 A deep reinforcement learning algorithm incorporating Bayesian optimization

The combination of deep reinforcement learning and Bayesian optimization not only inherits the dynamic decision-making ability and efficient computational advantages of deep reinforcement learning, but also dynamically adjusts the hyperparameters by guiding the hyperparameter search through the probabilistic model of Bayesian optimization, which further strengthens the algorithm’s global optimization ability. This fusion not only overcomes the problem that traditional heuristic algorithms are prone to fall into local optimum and computational inefficiency, but also significantly improves the efficiency and accuracy of hyper-parameter tuning through the adaptive sampling mechanism of the Bayesian framework, so that the algorithm combines both the generalisation ability of deep reinforcement learning and the intelligent exploratory characteristics of Bayesian optimization, and demonstrates stronger convergence and stability in complex decision-making problems.

#### 2.6.1 A proximal policy optimization algorithm incorporating Bayesian optimization

**Figure 2:**
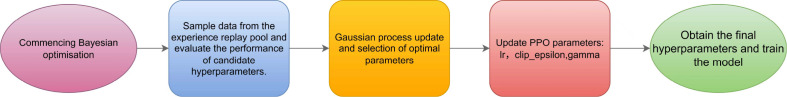
Flowchart of the two-tier optimization system.

A deep synergistic framework based on Bayesian optimization and proximal policy optimization (PPO) achieves dynamic adaptive training of forest management decision-making systems. The architecture combines the Bayesian optimiser into the training cycle of PPO through a periodic hyper-parameter tuning mechanism, forming a two-tier hierarchical optimization system: the upper Bayesian optimization module is based on Gaussian process modelling, and automatically searches for the optimal hyper-parameter combinations (including the learning rate, the policy trimming range and the discount factor) after every N policy iterations; and the lower PPO body continuously optimises the logging decision-making strategy using the tuned parameters. The Bayesian optimization process first randomly samples several sets of initial hyperparameters (learning rate lr, policy clipping coefficient clip epsilon, discount factor gamma) from the experience recall pool and evaluates the performance of the candidate parameters. Based on these initial data, it constructs a Gaussian process agent model update and selects the optimal hyperparameters. Subsequently, the configuration of the PPO algorithm is updated based on the optimal hyperparameter combinations predicted by the Gaussian process agent model. The detailed flow of this two-layer optimization system is shown in **Figure 2**.

**Figure 3:**
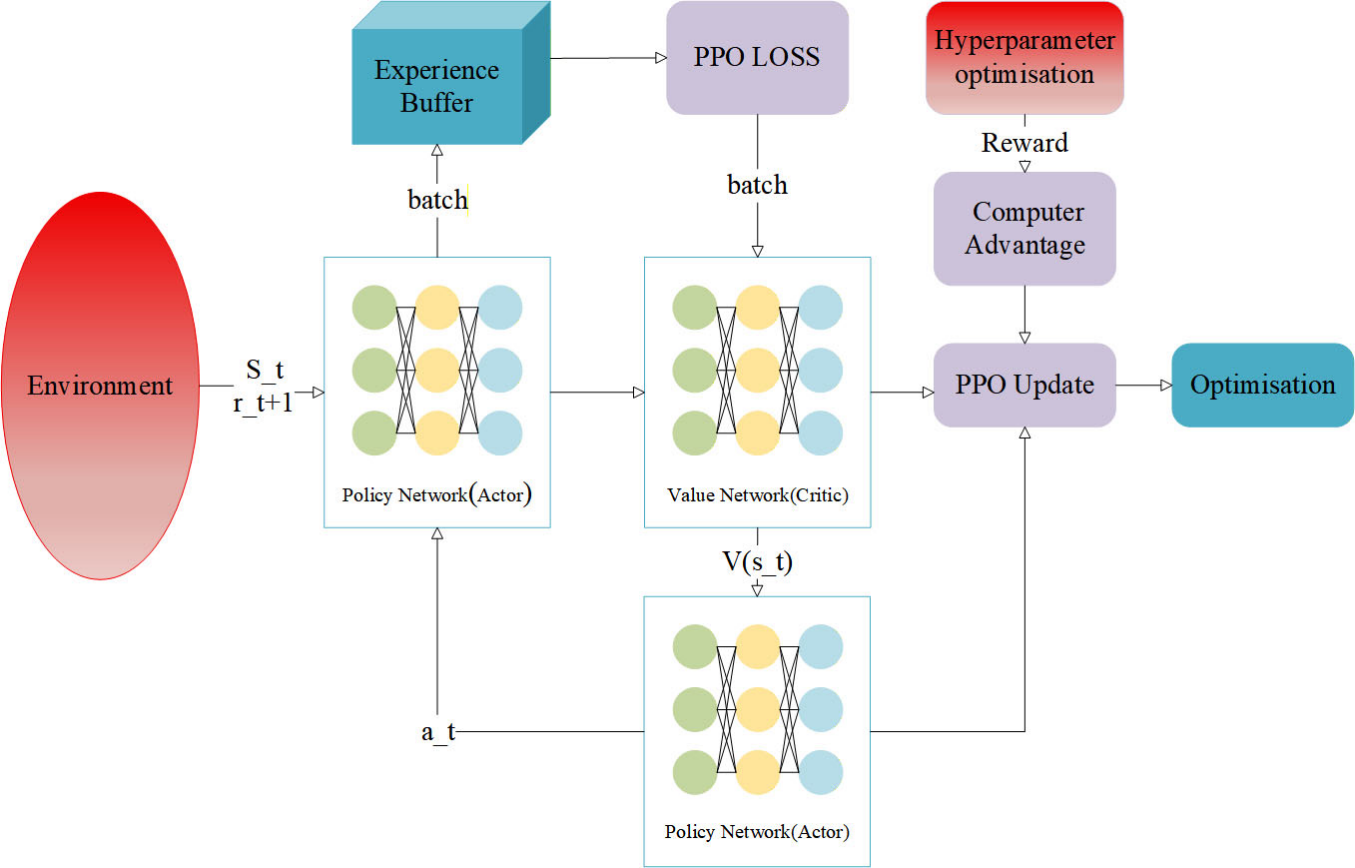
Structure of the PPO-BO model.

In order to efficiently solve multi-objective optimization problems with stand structure, this study proposes a proximal policy optimization (PPO-BO) algorithmic framework based on Bayesian optimization. The framework takes the PPO algorithm as the core decision engine and innovatively introduces a Bayesian optimization module for automated, offline optimization search of key hyper-parameters (e.g., learning rate, cropping range) before training, aiming to improve the algorithm’s convergence speed and final optimization effect. The PPO algorithm adopts a parallel environment sampling and prioritised empirical playback pool to accelerate the data collection, and constructs a strategy network and a value network that contain a dual architecture, both of which are independent of the feature extraction layer (Schulman et al., 2017; Yu et al., 2022). The state *S* in the PPO network is a one-hot coded vector of length 100 indicating the current progress of the selection process. The strategy is updated using a truncated objective function *L^CLIP^*(*θ*) (Schulman et al., 2017; Yu et al., 2022), and the value function *V* (*s_t_*) (Schulman et al., 2017) is updated by minimising the mean-square error (MSE) between the predicted value and the first-order temporal difference objective value. Bayesian optimization is used to optimise key hyperparameters (e.g., learning rate, trimming range, discount factor) offline before training, with expected improvement (EI) as the acquisition function.Actor network outputs Bernoulli distribution parameters, sampling to generate felling decisions and preserve strategy randomness. In addition, an entropy regularisation term is added to the objective function to enhance exploration. Compared with the traditional DQN, the PPO algorithm significantly improves the stability of reinforcement learning through the trust domain mechanism, the core of which lies in constraining the magnitude of the policy update to avoid the performance crash due to the single-step update being too large. This is achieved in two ways: first, probability ratio pruning, which forces the action probability ratios of the old and new strategies to fall within the interval [1 − *∈*,1 + *∈*], suppressing aggressive parameter updates. The second is adaptive KL scatter penalty, which dynamically adjusts the learning rate to control strategy differences. Compared with the DQN algorithm, which relies on the greedy selection of Q-value, it is not easy to fall into the local optimum. The DQN algorithm only supports discrete action space, while the PPO algorithm supports both discrete and continuous action space, which can dynamically adjust the proportion of random harvesting, while the DQN algorithm needs to pre-set the harvesting intensity. Experiments show that the algorithm demonstrates significant advantages in training efficiency and optimization effect (the highest improvement of objective function is 31.87%), which provides an efficient and reliable solution for multi-objective optimization of forest stand structure. The structure of the PPO model is shown in **Figure 3**.

Where the truncated objective function is expressed as Equation 18:

(18) $$L^{CLIP}\left(\theta \right)=E_{t}\left[min\left(r_{t}\left(\theta \right){\hat{A}}_{t},clip\left(r_{t}\left(\theta \right),1-\in ,1+\in \right){\hat{A}}_{t}\right)\right]$$

Where *θ* denotes the current strategy parameter; *r_t_*(*θ*) is equal to $\frac{\pi_{\theta }\left(a_{t}/s_{t}\right)}{\pi_{\theta_{odd}}\left(a_{t}/s_{t}\right)}$ denotes the probability ratio of the current strategy to the old one; ${\hat{A}}_{t}$ denotes the superiority or inferiority of the action *a_t_* with respect to the average strategy; and *∈* is a truncation hyperparameter to limit the maximum magnitude of the strategy update. *clip*(*r_t_*(*θ*),1 − *∈*,1 + *∈*) is the stage function that limits the probability ratio to the interval [1 − *∈*,1 + *∈*], preventing a single update from being too large. This truncation mechanism is central to the stability of the PPO algorithm, and effectively prevents destructive policy oscillations while encouraging policy updates in a favourable direction.

The value function is used to estimate the expectation of the sum of rewards obtained at each future step according to the strategy *π_θ_* in state *s_t_*. In this study, the value function *V* (*s_t_*) is theoretically defined as Equation 19:

(19) $$V\left(s_{t}\right)=E_{\pi_{\theta }}[\sum_{t=0}^{\infty \infty }\;\gamma^{l}r_{t+l}|s_{t}]$$

Where *V* (*s_t_*) denotes the cumulative discounted reward expected to be earned in the future under the current state *s_t_*; $E_{\pi_{\theta }}$ denotes the weighted average *π* representation strategy over all possible actions and state transfers under the strategy *π_θ_*; *γ* denotes the discount factor used to weigh the importance of the current reward against the future rewards; and *r_t_*_+_*_l_* denotes the instantaneous reward earned from the *t*+*l*th step.

In practice, we use the time-difference (TD) method to estimate this expectation and train the value network by minimising the mean-square error loss so that it can accurately assess the long-term value of different forest stand states. The expression is given below in Equation 20:

(20) $$V\left(S_{t}\right)\approx r_{t}+\gamma V\left(s_{t+1}\right)$$

Where *V* (*s_t_*) denotes the future reward expectation of the current state, estimated by the Critic network; *r_t_* denotes the immediate reward obtained after the current action is taken; *γ*, as above, measures the importance of the future reward; and *V* (*s_t_*_+1_) is the future reward expectation of the next state, again predicted by the Critic network.

#### 2.6.2 Optimization of stand structure solution

**Figure 4:**
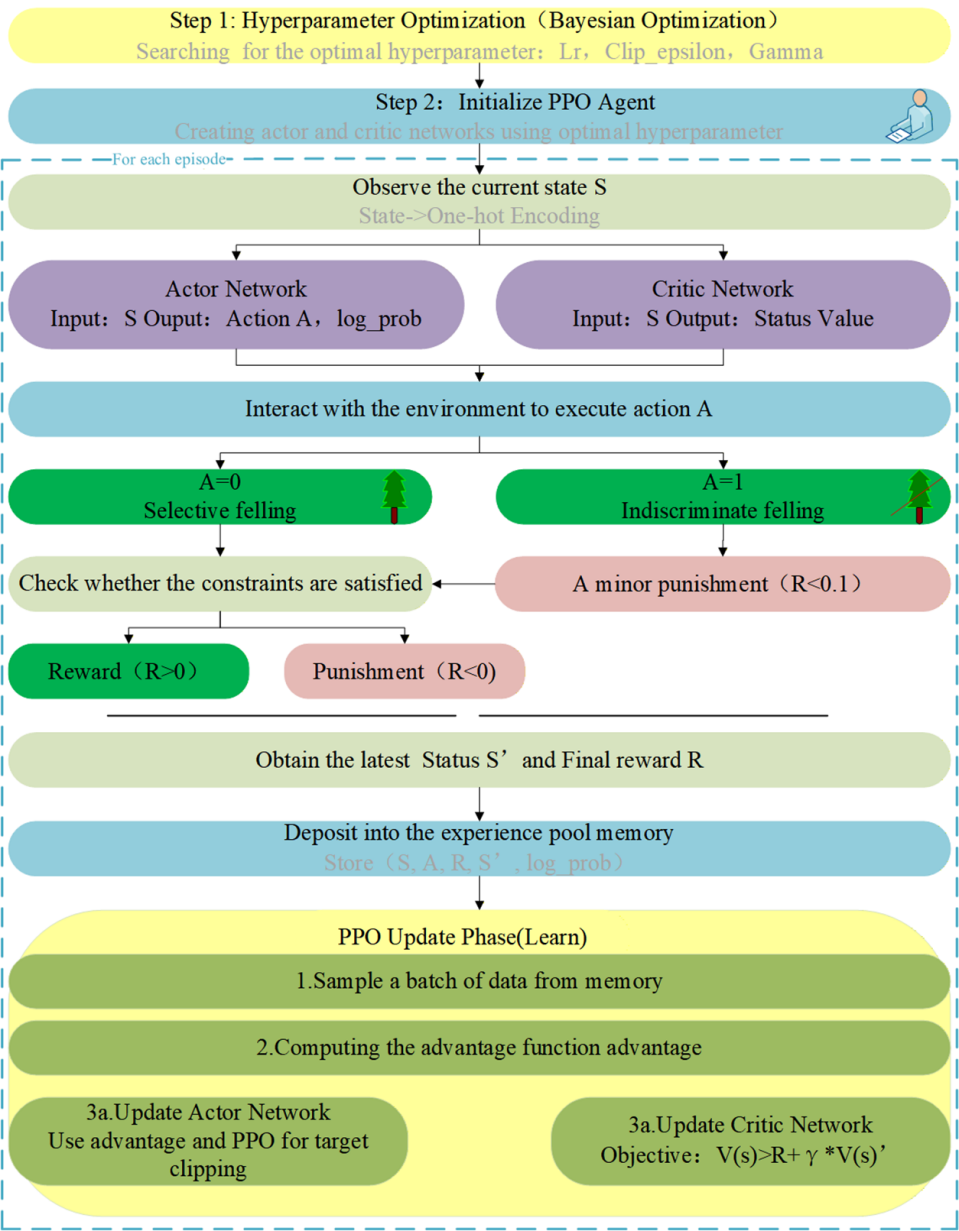
Flowchart of the PPO-BO algorithm for optimising stand structure.

This study abstracted the complex forest harvesting selection problem as a sequential decision-making process in reinforcement learning. In this framework, the action of an intelligent body corresponds to whether or not to perform a logging operation. After each decision, the environment generates a candidate harvesting set based on a predefined proposed logging selection strategy (e.g., random selection, multiparameter comprehensive evaluation, etc.) and simulates the harvesting. Subsequently, the merits of the decision are quantified by calculating the change in the objective function value of the optimised stand, which is converted into a reward signal to be fed back to the intelligences to guide the optimization of subsequent strategies. By generating a set of candidate trees based on a predefined method for determining the trees to be removed, and calculating the change in the objective function value of the stand by simulating the harvesting operation, the optimization effect is evaluated, and a reward mechanism is designed accordingly to guide the intelligent body’s subsequent decision-making. In the training and execution process, the intelligent body continuously accumulates the experience of interacting with the environment, the Actor network samples action A and outputs a continuous determination of the harvesting ratio during execution and training, outputs the action and log prob according to the state *S*, and interacts with the environment according to the action to get the reward and the next state. After completing the training and execution process, *state*, *action*, *reward*, *next_s_tate*, log prob are stored in the experience playback buffer.The Critic network evaluates the goodness of the state based on the current state value *V* (*s_t_*) during training and is used to compute the Advantage function *AdvantageA*(*s,a*), which is then computed by the intelligence. The body updates and optimises the intelligence based on *AdvantageA*(*s,a*) and the parameters in the buffer. By regularly updating the parameters in the memory, the intelligent body can gradually adapt to the dynamic changes of the forest stand structure and make better harvesting decisions. Eventually, the mechanism can guide the intelligent body to find the harvesting plan that maximises the cumulative reward, which significantly improves the effectiveness and efficiency of the optimization of stand structure. The flow chart of PPO for solving stand structure is shown in **Figure 4**. The specific implementation details are outlined in **Algorithm 1** and **Algorithm 2**.

Algorithm 1 **Hyperparameter optimization.**

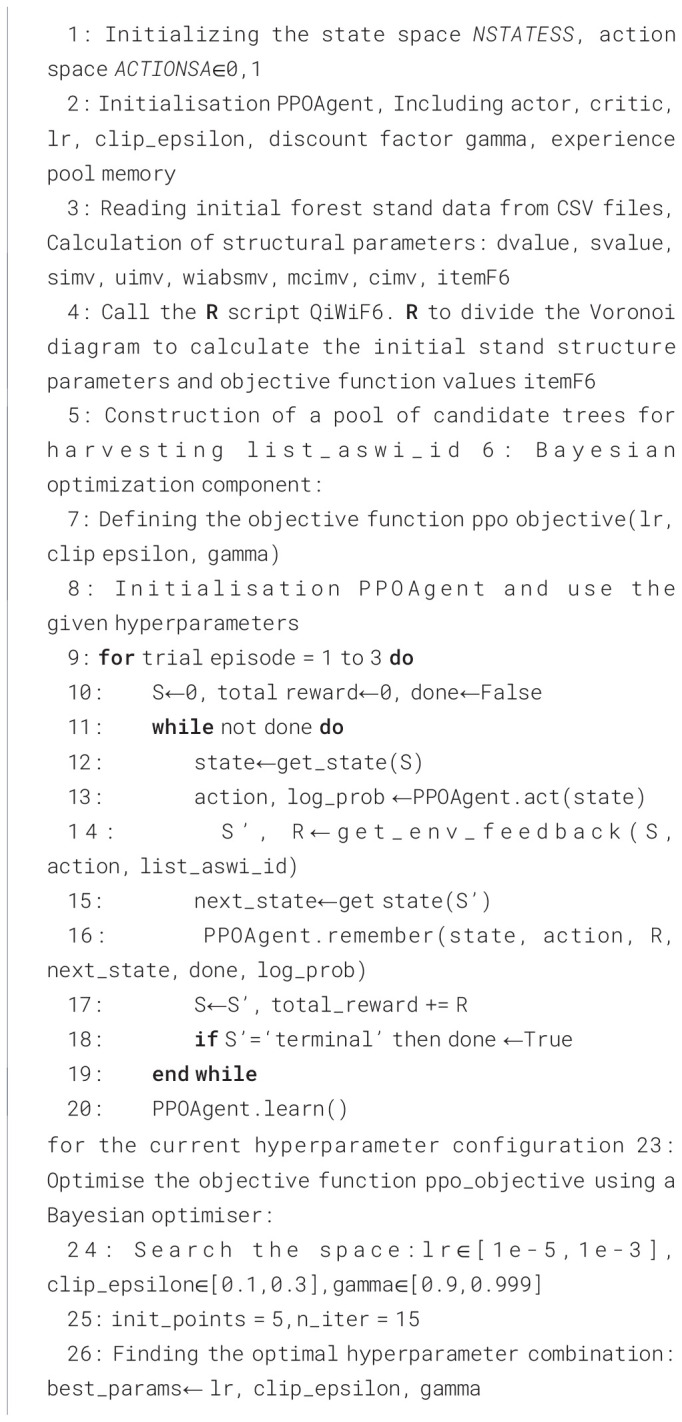

Algorithm 2 **PPO algorithm for stand structure optimization.**

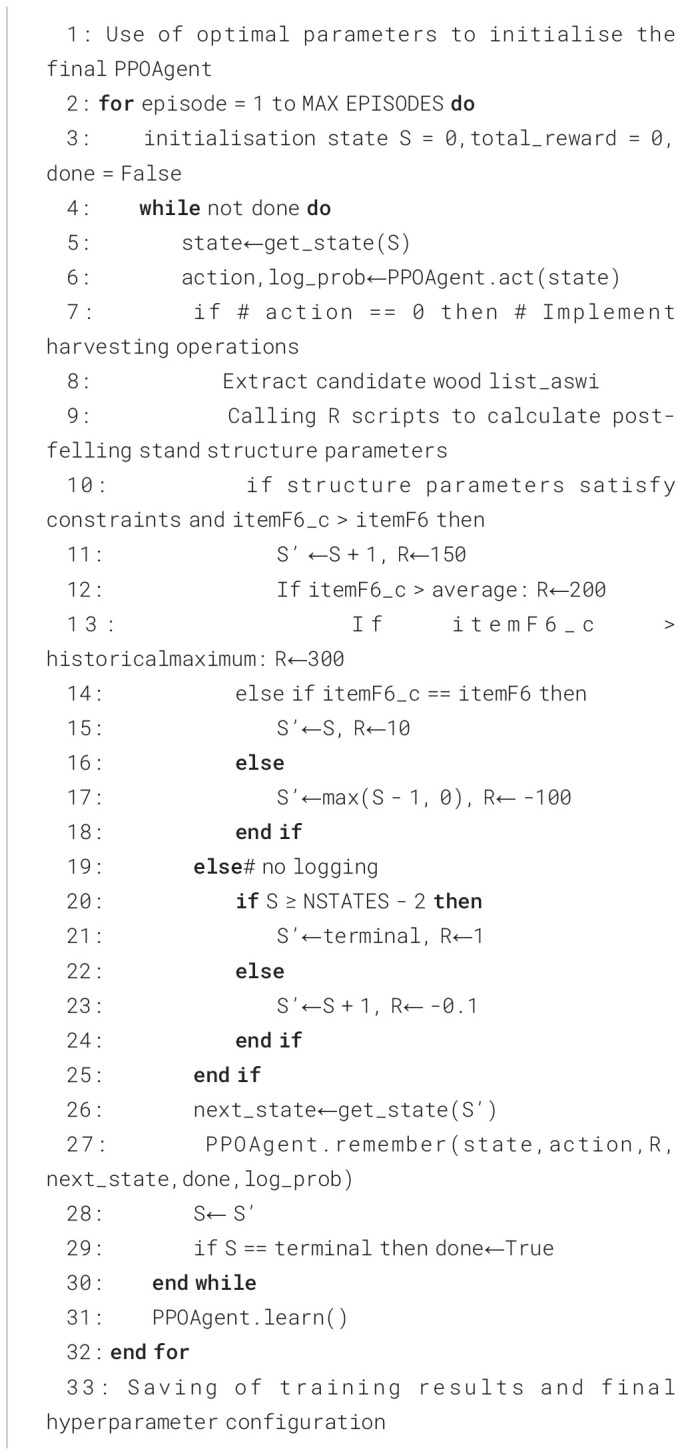

### 2.7 Optimization strategy and experimental setup

#### 2.7.1 Optimization strategy

In order to evaluate the effectiveness of deep reinforcement learning algorithms in the practical application of multi-objective optimization of forest stand structure, a systematic comparative experimental scheme was designed in this study. Five standard plots with different densities and stand conditions were selected as experimental objects, and the performance differences of different optimization schemes were investigated through simulated harvesting experiments. Six combinations of optimization schemes were set up, including the cross-combination of three proposed log removal methods (based on different selection criteria) and two solution algorithms (Deep Reinforcement Learning DQN and Proximal Policy Optimization PPO). Through the multi-dimensional comparative analysis, we focused on the influence of the proposed log removal selection strategy on the model optimization effect, as well as the solution performance of different algorithms in the forest stand structure optimization problem. The six optimization combinations are shown in **Table 2**.

**Table 2:** Comparative performance of two algorithms across three selection schemes.

Selection methods for prescribed cutting trees	DQN	PPO-BO
randomly selected	M1	M2
Integrated multi-parameter evaluation	M3	M4
Optimization of spatial patterns	M5	M6

#### 2.7.2 Experimental parameter settings and algorithm configuration

In this experiment, the proximal policy optimization (PPO) algorithm was used to optimize the stand structure, and intelligent decision-making was achieved through the Actor-Critic framework. The algorithm parameter setting is divided into two phases: in the basic parameter setting phase, we constructed the Actor and Critic model containing a 3-layer fully-connected network (the hidden layer is fixed to 24 dimensions), set the capacity of the empirical playback pool to 10,000 transitions data, and the state space was abstracted as a discrete sequence of 0-100, with each round of iteration starting from the initial state until the termination state; The Bayesian optimization phase then dynamically adjusts the core hyperparameters, including the learning rate *lr* (1e-5 to 1e-3), the policy clipping range clip epsilon (0.1-0.3), and the discount factor *γ* (0.9-0.999), and determines the optimal parameter combinations through 15 rounds of Bayesian optimization. The optimised parameter configurations are used for final training, the full empirical batch is used for strategy updating, action selection is based on Gaussian distribution sampling (outputting mean and standard deviation), and the generalised advantage estimation (GAE) method is used for advantage estimation. In order to compare the performance of PPO and DQN, PPO and DQN are set with two identical initial parameters (*γ* = 0.9, *lr* = 0.01). In particular, PPO ensures training stability through importance sampling and a policy clipping mechanism (clip epsilon=0.2), while incorporating Bayesian optimization for adaptive tuning of the hyperparameters. Experiments show that this configuration can effectively improve the optimization effect of forest stand structure optimization indexes (e.g. depression attainment rate and objective function itemF6 value) while ensuring the convergence of the algorithm. The experimental parameter settings are shown in **Table 3**.

**Table 3:** Experimental parameter setting.

Method	Hyperparameter name	typology	Search range/candidates	Bayesian optimization constraints
PPO-BO	Learning rate (*lr*)	continuous value	[1e-5,1e-3]/0.01	logarithmic transformation
	Discount factor (*γ*)	continuous value	[0.9,0.999]/0.9	–
	batch size	fixed value	32	–
	clip epsilon	continuous value	[0.1,0.3]/0.2	logarithmic transformation

## 3 Analysis of results

### 3.1 The impact of gradient constraints on the optimization performance of the PPO-BO algorithm and a comparison with DQN

#### 3.1.1 The effect of slope constraints on candidate trees for felling

In practical forest management, trees located on steep slopes play a crucial role in soil and water conservation; therefore, imposing protective restrictions on trees in high-gradient areas is of considerable ecological significance. In this study, statistics were compiled for trees situated on slopes exceeding 45° across five sample plots. The number of trees excluded under the slope constraint, along with their respective proportions, are presented in **Table 4**. As shown, Plot P2 exhibited the highest exclusion rate (20.88%), consistent with its pronounced topographic variability, whereas Plot P4, with the lowest average slope (5.10°), had the smallest proportion of excluded trees (3.17%). The introduction of slope constraints effectively safeguards trees in steep areas that are critical for soil and water conservation, while simultaneously reducing the pool of candidate trees for felling, thereby directly influencing the optimization process.

**Table 4:** Total number of exclusions under different slope constraints.

Sample plot	Total number of plants	Excluded strains	Exclusion rate (%)
P1	1291	82	6.35%
P2	1250	261	20.88%
P3	421	69	16.38%
P4	378	12	3.17%
P5	1132	117	10.33%

#### 3.1.2 A comparison of the optimization performance of the PPO-BO algorithm with and without slope constraints

To quantitatively evaluate the contribution of the slope constraint to optimization performance, comparative experiments were conducted under the same algorithmic framework (PPO-BO), with and without the slope constraint. The results are presented in **Table 5**. The results showed that, after incorporating the slope constraint, the PPO-BO algorithm achieved higher optimal objective function values across all five plots compared to the unconstrained case. This indicates that, rather than diminishing optimization performance by restricting the candidate set, the slope constraint effectively guides the algorithm towards ecologically appropriate felling decisions. As a result, the proportion of ineffective searches is reduced, enabling the policy network to explore a more meaningful decision space and thereby improving overall optimization quality. Plot P2 showed the greatest increase, reaching 1.61%; this is due to the complex topography of Plot P2 itself, where a significant proportion of the trees are located on slopes steeper than 45 degrees. The improvement observed in Plot P4 is relatively modest, likely due to its low slope, which results in only a small number of trees being excluded; consequently, the difference between the constrained and unconstrained scenarios is minimal. This observation further supports the underlying mechanism of the slope constraint.

**Table 5:** Comparison of objective function values with and without constraints.

Sample plot	Starting value	PPO-BO (Unrestricted)	PPO-BO (constrained)	Improvements brought about by constraints
P1	0.3501	0.4454	0.4475	0.47%
P2	0.3799	0.4995	0.5075	1.61%
P3	0.3982	0.5092	0.5146	1.06%
P4	0.3344	0.5112	0.5133	0.41%
P5	0.4294	0.5187	0.5255	1.29%

#### 3.1.3 Comparison of quantitative performance of PPO and DQN in stand structure optimization

In this study, simulated logging experiments were conducted on five sample plots (P1-P5) using six optimization schemes, and the results showed that each scheme significantly improved the objective function value. As can be seen from **Table 6**, the objective function values of each sample plot were improved to different degrees after optimization, and the objective function values of sample plots P1 to P5 were improved from 0.3501, 0.3799, 0.3982, 0.3344, 0.4294 to a maximum of 0.4475, 0.5075, 0.5146, 0.5133, 0.5255, with a maximum of 31.87%. Among them, sample site P4 had the most outstanding performance, with an increase of 53.50% in the objective function value from 0.3344 to 0.5133; the rest of the sample sites had an increase of between 12.76% and 29.23%. It is particularly noteworthy that the PPO algorithm outperformed the DQN optimization in all sample plots, with an average improvement of 13.23% over DQN.

**Table 6:** Objective function values before and after simulated felling for each optimization scenario.

Methods	P1	P2	P3	P4	P5	Average value	Enhancement
starting value	0.3501	0.3799	0.3982	0.3344	0.4294	0.3784	
M1	0.4421	0.4973	0.5051	0.4947	0.5057	0.4890	29.22%
M2	0.4475	0.5075	0.5146	0.5133	0.5122	0.4990	31.87%
M3	0.4194	0.4616	0.4612	0.4393	0.4834	0.4530	19.17%
M4	0.4359	0.4682	0.4934	0.4780	0.5255	0.4802	26.90%
M5	0.4161	0.4630	0.4583	0.4679	0.4963	0.4604	21.67%
M6	0.4254	0.4793	0.4644	0.4974	0.4975	0.4728	24.94%

**Figure 5:**
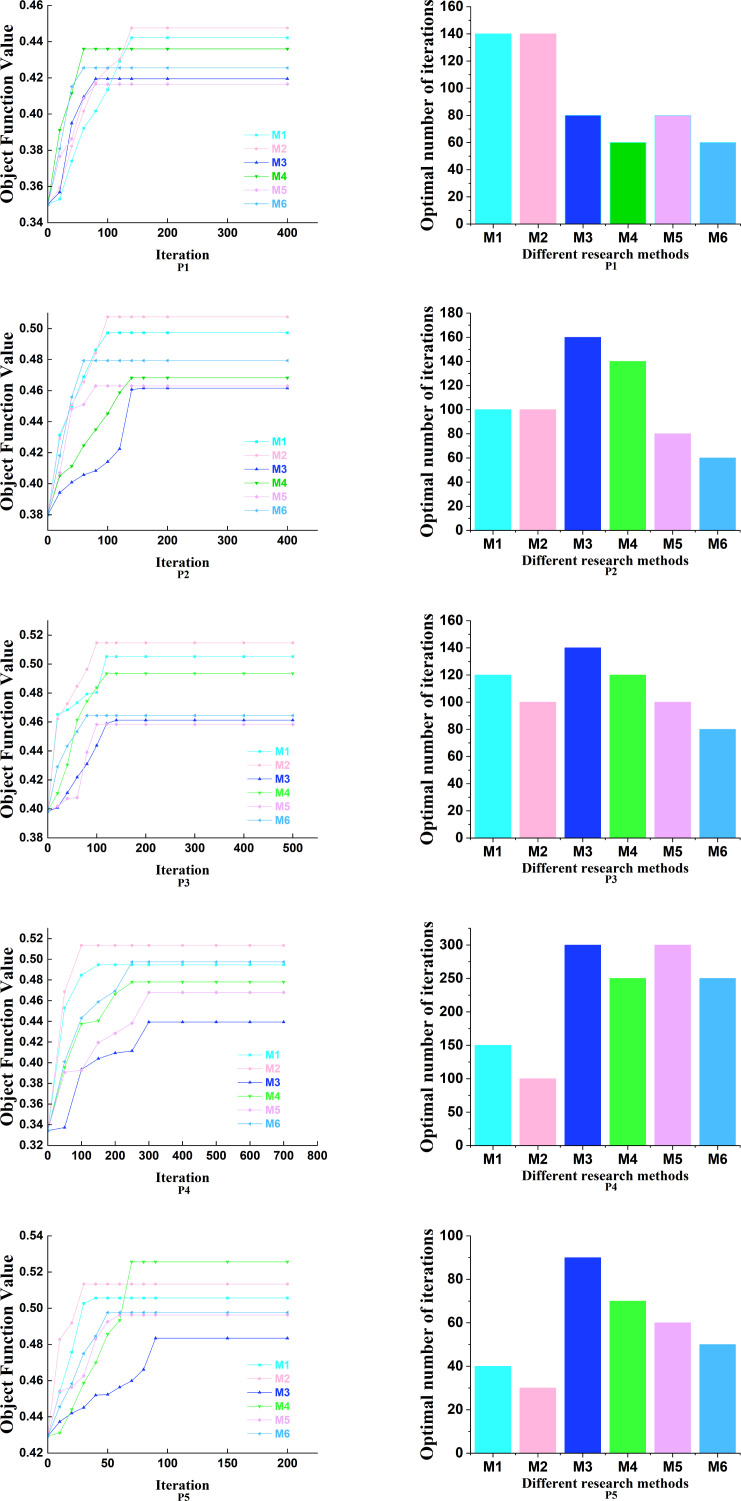
A comparison of the number of iterations required for convergence and the optimal objective function values for different optimization strategies across various training datasets.

**Figure 6:**
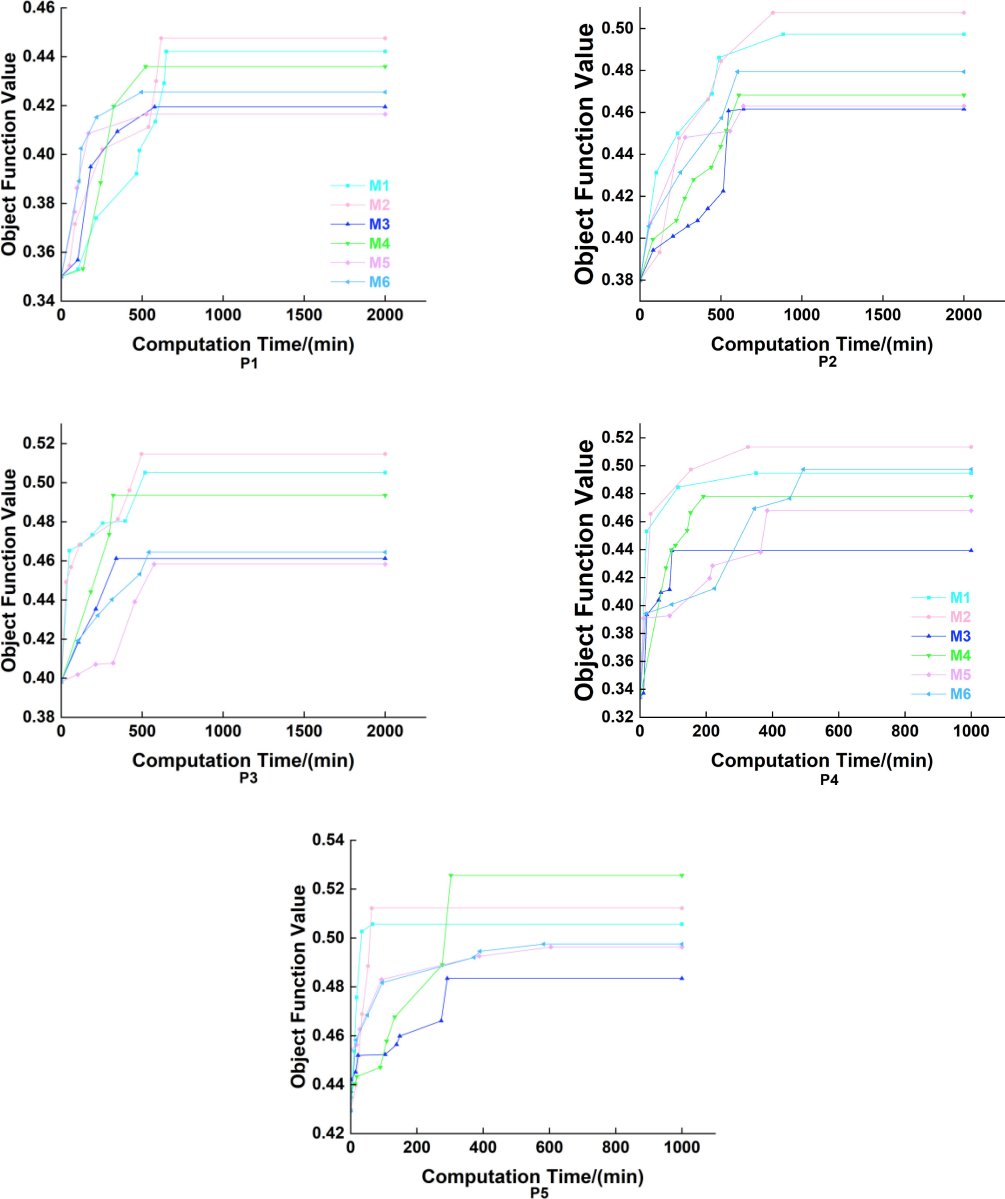
Objective function value versus computation time under different selection schemes.

In terms of convergence performance and algorithm time, the PPO algorithm showed obvious advantages. As shown in **Figure 5**, PPO had faster convergence speed and more stable optimization process in all samples, and its average number of convergence iterations was about 35% less than that of DQN. Especially in samples P3 and P4, PPO converged significantly faster, and only 70% of the number of iterations of the DQN algorithm is needed to achieve similar optimization results. And the computational efficiency of the PPO algorithm was significantly better than that of the DQN algorithm in solving the optimal objective function value, which is visually reflected in the experimental results in **Figure 6**. This difference fully reflects the superiority of the PPO algorithm in exploring-exploiting balance, which can deal with the complex constraints of forest stand spatial structure more efficiently.

### 3.2 The impact of different proposed thinning schemes on the multi-objective optimization of stand structure

**Figure 7:**
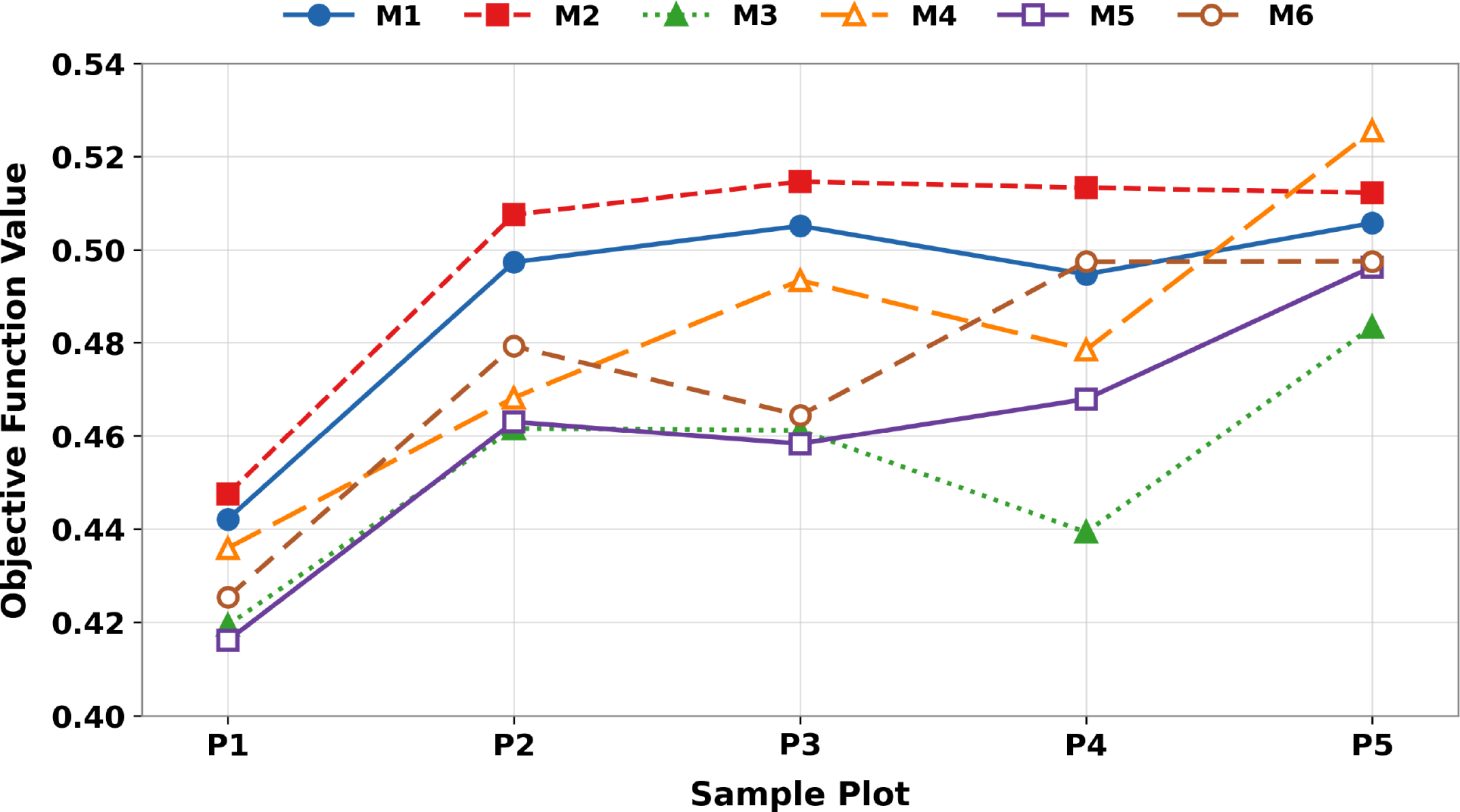
Effect of different proposed logging on the optimal objective function value of the algorithm.

As shown in **Figure 7**, the effect of different proposed logging on the optimal objective function value of the algorithm is clearly indicated, and the PPO-BO algorithm has the optimal optimization effect and higher objective function value than the DQN algorithm in the combination of random selection and multi-parameter comprehensive evaluation and selection schemes. In the DQN algorithm, the optimal optimization effect of all the samples is concentrated in the random selection scheme, while the PPOBO algorithm achieves the optimal objective function value of the P5 sample in the multi-parameter comprehensive evaluation and selection scheme, which is higher than that of the random selection scheme, with the improvement of 22.38%, which is significantly higher than that of the DQN algorithm, which is 12.57%. Secondly, in the M4 and M6 scenarios, the impact of the proposed logging scheme is more significant than that of the DQN algorithm, and the objective function values of each sample site are improved to different degrees, which indicates that the PPO algorithm is more suitable than the DQN algorithm for these three proposed logging schemes. This study will explain this phenomenon in the following ways.Firstly, from the perspective of search space characteristics, both the multi-parameter comprehensive evaluation strategies (M3/M4) and the spatial pattern optimization strategies (M5/M6) rely on deterministic rules to pre-screen candidate trees for felling. This process effectively introduces additional prior constraints external to the reinforcement learning decision space, thereby restricting the range of feasible actions. When these constraints are not fully consistent with the true distribution of optimal solutions, they may impede the algorithm’s ability to explore globally optimal regions. In contrast, the random selection strategy imposes no structural bias on candidate trees, thus preserving the full search space for the PPO-BO algorithm. This enables the model to identify superior harvesting combinations through reward-driven exploration, which is more consistent with the fundamental mechanism of PPO-based optimization.

Secondly, the multi-parameter integrated evaluation strategy combines several spatial structural indicators—such as mingling, size differentiation, and competition indices—using an equal-weight scheme. However, these indicators exhibit inherent non-linear interactions and potential conflicts. Under certain stand conditions, prioritising the removal of trees with high size differentiation may disrupt a spatial pattern that has already approached a desirable random distribution, resulting in trade-offs among objectives and weakening the effectiveness of optimization across multiple dimensions. By contrast, random selection strategies are not subject to such structural constraints and therefore provide greater flexibility for the optimization process.

It is important to note, however, that the apparent advantages of random selection strategies do not diminish the underlying compatibility between the PPO-BO framework and structured strategies. Under the multi-parameter evaluation strategy (M4), PPO-BO achieved the highest stand structure index (0.5255) among all schemes in plot P5. Moreover, the improvement achieved by PPO-BO relative to DQN under the M4 scheme (26.90% vs. 19.17%) was substantially greater than the corresponding difference under the M2 scheme (31.87% vs. 29.22%). This indicates that PPO-BO demonstrates stronger algorithmic compatibility with structured strategies, with its advantages more fully realised within such frameworks.

This observation further suggests that the higher absolute improvement values associated with random selection strategies are largely attributable to their lower initial baseline—reflecting greater optimization potential in an unconstrained state—rather than an inherent superiority of the strategy design itself.

### 3.3 Analysis of the spatial structure after the proposed selective logging

**Figure 8:**
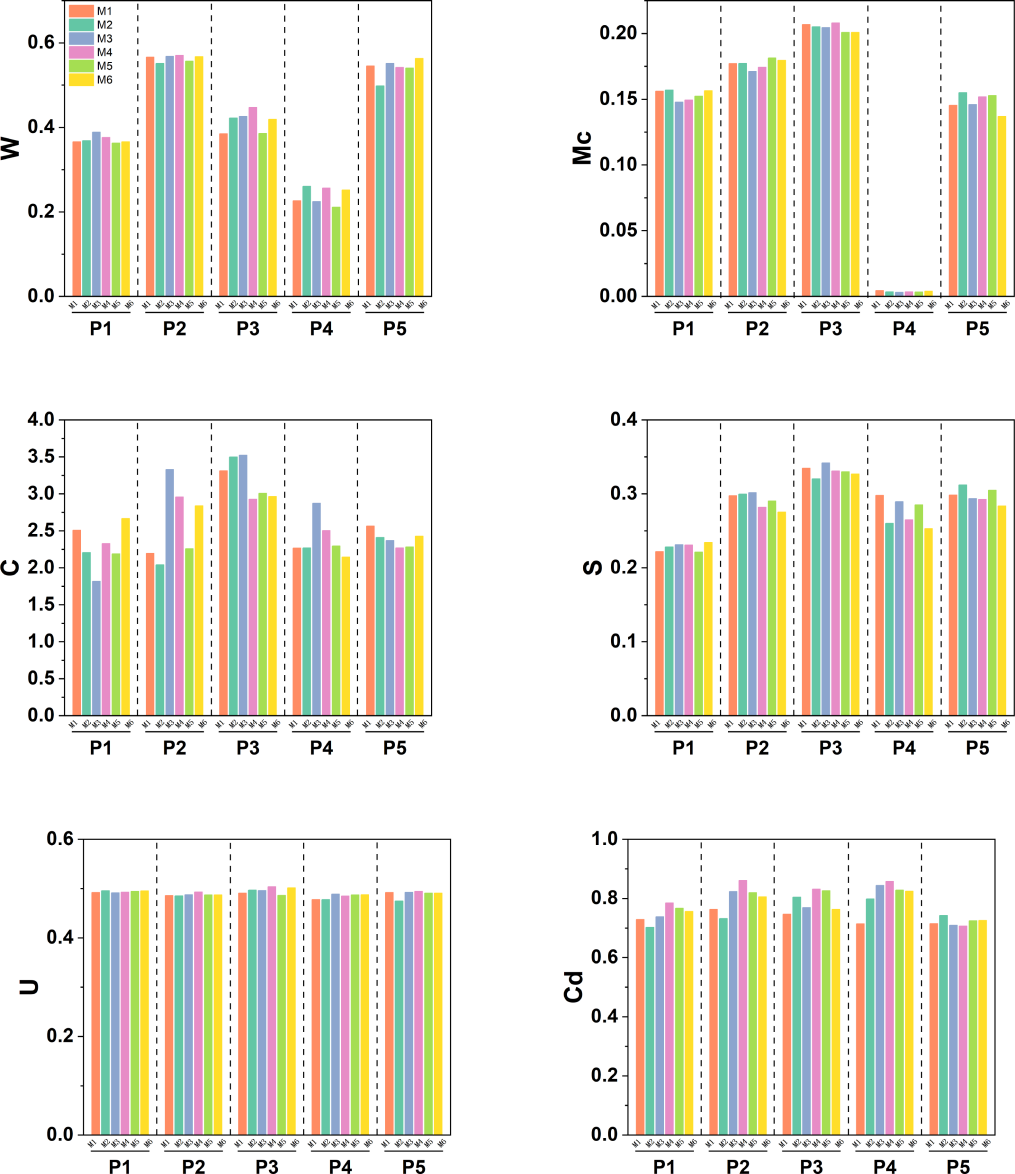
Comparative analysis of stand structure indicators under different management schemes.

In this study, we used the Voronoi diagram method to calculate the maximum objective function value *L_i_*, selectively logged between 0 and 35% of the intensity, and quantitatively analysed the two algorithms based on three different proposed logging scenarios to determine the parameters of the stand structure, see **Figure 8**. The spatial structure indices for the various stands following optimization are shown in **Table 7**. In terms of angular scale, following selective cutting optimization, the mean angular scales of plots P1, P3, P4, and P5 all converged towards the theoretical range of random distribution [0.475, 0.517]. Among these, plot P5 exhibited the most pronounced improvement, with its mean angular scale closest to the median value of the random distribution (0.496). Although the remaining plots displayed different adjustment trends due to variations in their initial spatial configurations—with some showing increases and others decreases in angular scale—all changes consistently moved towards the random distribution range. This indicated that selective felling effectively enhanced the horizontal spatial distribution pattern of the stands. In terms of overall mixing degree, all plots demonstrated varying levels of improvement following optimization, with increases ranging from 0.49% to 35.25%. Plot P4 showed the greatest increase, reaching 50.0%, which is closely associated with its initially low species mingling. Generally, the lower the initial mixing degree, the greater the potential for improving species spatial configuration through selective felling, resulting in a more pronounced optimization effect. Regarding the canopy competition index, all plots exhibited reductions after optimization, with decreases ranging from 9.3% to 40.16%. Plot P2 showed the largest decline, reaching 40.16%. This reduction indicates that selective felling effectively alleviated spatial competition among trees, thereby improving access to light and nutrients for individual trees and promoting the subsequent growth of retained individuals. With respect to the forest stratification index, all plots achieved varying degrees of improvement, with increases ranging from 0.29% to 10.95%. Although the magnitude of improvement in some plots was relatively modest, the overall trend suggests that appropriate harvesting interventions facilitated the differentiation and development of vertical stand structure, promoting the transition towards a multi-layered forest by releasing growth space for understory trees. In terms of canopy closure, the optimised values for all plots remained within the range of 0.72 to 0.86, satisfying the constraint that canopy closure should not fall below 0.7. This demonstrates that, while effective harvesting interventions were implemented, continuous canopy coverage was successfully maintained, ensuring that the stand remained in a healthy condition approaching natural closure levels.Regarding the size ratio, post-optimization reductions across all plots were relatively stable, with only minor variation. This outcome does not indicate insufficient optimization; rather, it suggests that the stand continues to maintain a moderate level of competition among individuals, with dominant trees effectively retained. Such a structure is beneficial for sustaining long-term stand productivity and supporting the ongoing process of natural regeneration.

**Table 7:** Forest stand structure indicators for various plots.

Plot	State	Angular scale	Full hybridization	Canopy competition index	Stratification index	Size ratio	Objective function	Increase (%)
P1	Before After	0.3630 0.3737	0.1459 0.1569	2.7130 2.2053	0.2205 0.2284	0.4960 0.4959	0.3501 0.4475	27.82
P2	Before After	0.5612 0.5516	0.1693 0.1773	3.4130 2.0420	0.2772 0.2999	0.4906 0.4853	0.3799 0.5075	12.76
P3	Before After	0.3867 0.4221	0.1990 0.2052	4.0990 3.4991	0.3286 0.3377	0.4965 0.4952	0.3982 0.5146	29.23
P4	Before After	0.2103 0.2683	0.0028 0.0042	3.1363 2.2689	0.2846 0.2747	0.4930 0.4779	0.3344 0.5133	53.50
P5	Before After	0.5551 0.5422	0.1443 0.1518	2.8202 2.2707	0.2882 0.2926	0.4950 0.4947	0.4294 0.5255	22.38

## 4 Discussion

The optimization of stand structure remains a central challenge in sustainable forest management. Developing scientifically sound quantitative models of stand structure and designing efficient, tailored solution algorithms are therefore critical issues that remain key issues requiring urgent attention in this field. In the construction of stand structure models, both spatial and non-spatial structural parameters serve as core variables, with the value of the objective function being positively correlated with the degree of optimization—i.e., higher values indicate that the stand condition is closer to an ideal state. In this study, the stand structure quantification model proposed by Zhao et al. (2024) was adopted to facilitate a comparative analysis of two algorithms. This model incorporates five key spatial structural parameters—angular scale, size ratio, overall mixing degree, stand heterogeneity index, and canopy competition index—to characterise stand structure (Zhao et al., 2024). However, the design of solution algorithms for such models faces challenges including high computational demands and low efficiency. Neither traditional ecological methods nor conventional intelligent optimization algorithms can adequately overcome the limitations of low efficiency and weak solution capability in stand structure optimization. In recent years, with the rapid development of artificial intelligence, reinforcement learning has emerged as a promising approach for addressing multi-objective optimization problems across various domains. It offers the ability to adapt dynamically to changes in objective weights, constraints, and environmental states, thereby providing decision-making capabilities that are difficult to achieve using traditional mathematical programming and evolutionary algorithms (Malo et al., 2021; Chen and Chang, 2014). Reinforcement learning has already been applied in the field of stand structure optimization (Xuan et al., 2023; Zixuan et al., 2023; Dongsheng et al., 2020); however, it still suffers from issues such as solution instability and limited generalisation ability (Chenbo et al., 2018; Watkins and Dayan, 1992). To address these shortcomings, deep reinforcement learning has gained increasing attention. By integrating the representational power of deep learning with the decision-making framework of reinforcement learning, it demonstrates strong performance in handling high-dimensional inputs and complex decision-making tasks. Its core advantage lies in the automatic extraction of high-level features through deep neural networks, enabling more efficient policy optimization and improved performance in multi-objective scenarios. Although previous studies have applied deep reinforcement learning to stand structure optimization models, systematic comparisons of the adaptability and performance of different deep reinforcement learning algorithms within this context remain lacking, thereby constraining both theoretical guidance for algorithm selection and practical application outcomes.

At the level of harvesting constraint design, most existing studies on forest stand structure optimization have primarily focused on silvicultural constraints, such as harvesting intensity, canopy closure, and species diversity, while topographic and site factors have seldom been incorporated into the decision-making framework. However, slope is a critical topographic factor influencing soil erosion risk in forest ecosystems. On steep slopes, tree root systems play a vital role in stabilising the soil, and large-scale felling in such areas can readily induce soil erosion and even geological hazards such as landslides (He, 2025). Previous studies have demonstrated that, with increasing slope gradient, surface runoff velocity accelerates and soil erosion intensity rises significantly. Once the slope exceeds a certain threshold, the ecological risks associated with logging operations increase sharply (Sun et al., 2021; Elliot et al., 2018). Against this background, the present study incorporates slope as a key constraint in the harvesting strategy by excluding trees located on slopes greater than 45°from the candidate set for felling, thereby achieving a coordinated balance between optimization objectives and ecological safety requirements.

Experimental results indicated that the introduction of slope constraints did not compromise optimization performance by limiting the candidate tree set. On the contrary, after incorporating slope constraints, the PPO-BO algorithm achieved higher objective function values across all plots compared with the unconstrained scenario. This phenomenon can be interpreted from two perspectives. First, trees located on slopes exceeding 45° are typically concentrated in areas with less favourable terrain conditions, and their contribution to overall spatial structure optimization is relatively limited; therefore, excluding them has minimal adverse impact on the objective function. Second, the rational reduction of the candidate solution space decreases the proportion of ineffective exploration by the policy network, enabling the agent to focus its training on ecologically feasible decision subspaces, thereby improving convergence quality. It should be noted, however, that the use of a fixed threshold of 45° represents a simplification and introduces certain limitations in practical applications. In reality, ecological risks vary continuously with slope gradient rather than changing abruptly, and the effects of slope often interact with other environmental factors, such as aspect, soil type, and precipitation intensity (Simelane et al., 2024; Wang et al., 2023). Future research could therefore explore the development of continuous slope-based risk weighting functions, transforming slope constraints from a binary exclusion rule into a continuous penalty mechanism. Additionally, integrating multiple topographic variables derived from soil erosion models may further enhance the refinement and realism of site condition constraints.

This study conducted simulated thinning experiments on five circular plots of secondary *Pinus yunnanensis* forests in south-western China. In stand structure optimization, the decision variable space is inherently dynamic; as stand conditions change, spatial structural parameters evolve accordingly. To address this characteristic, Bayesian optimization was introduced to handle uncertainties in objective function and constraint modelling, adapt to variations in parameter space, and significantly improve the efficiency and robustness of solving dynamic multi-objective optimization problems. This approach is particularly well suited to complex forestry scenarios involving resource constraints and time-varying objectives. The dynamic tuning of hyperparameters further enables deep reinforcement learning models to converge more rapidly towards optimal policies, thereby reducing computational costs. In addition, there are fundamental differences between the two algorithms examined: the DQN algorithm is primarily suited to discrete action spaces, whereas the PPO algorithm can accommodate both discrete and continuous action spaces, with the latter being more appropriate for determining thinning intensity. Experimental results show that, after incorporating Bayesian optimization, the objective function values obtained using the PPO algorithm were consistently higher than those produced by the DQN algorithm across all plots. From the perspective of spatial structural characteristics, the maximum species mixing degree was 0.28, indicating a generally low level of species mixing. Plots P2 and P5 exhibited clustered distributions, while the remaining plots displayed more uniform spatial patterns. The mean diameter-at-breast-height ratio was 0.49, suggesting a relatively balanced size distribution and moderate competition intensity among trees, which may reflect favourable natural regeneration or effective management interventions. The mean competition index decreased from 3.11 to 2.56, indicating a reduction in competitive pressure among neighbouring trees. The mean canopy Lin’s index increased slightly from 0.268 to 0.279, reflecting modest but positive changes in vertical structural complexity. Overall, although the optimised stands remained relatively stable, their structural complexity was still limited. Comparative results across different simulated thinning strategies further indicate that the PPO algorithm consistently outperformed the DQN algorithm in terms of stand structure indices. Notably, improvements were most significant under the M4 and M6 schemes. Under the M4 scheme, the average increase in the objective function value reached 26.90%, which is 7.7% higher than that achieved by the DQN algorithm; moreover, the highest stand structure index (0.5255) was observed in plot P5. Under the M6 scheme, the average improvement reached 24.94%, exceeding that of the DQN algorithm by 3.27%. Even under random selection schemes, the improvement surpassed that of the DQN algorithm (29.22%), reaching 31.87%. These results indicate that the PPO-based reinforcement learning approach exhibits stronger adaptability and effectiveness in multi-objective stand structure optimization, particularly under specific thinning strategies.It is noteworthy that, although random selection yielded the largest absolute improvement, the PPO algorithm also demonstrated stable and effective performance under the M4 and M6 strategies. This suggests that the PPO algorithm, when combined with Bayesian optimization for hyperparameter tuning, is well suited to these thinning approaches. Nevertheless, this study considered only three thinning strategies and a single site factor, which represents a limitation. Future research should incorporate a wider range of thinning methods, site conditions, and stand structure quantification models to further evaluate and enhance the applicability of deep reinforcement learning in this domain. Finally, this study provides a direct comparison of two deep reinforcement learning algorithms, demonstrating that policy gradient–based methods can more effectively adjust harvesting strategies in response to dynamic stand growth processes, whereas value-based methods require frequent manual parameter tuning. These findings highlight the superior adaptability of the PPO-BO framework in stand structure optimization.

## 5 Conclusions

Based on simulated thinning trials conducted in five circular plots of secondary *Pinus yunnanensis* forests on the eastern slope of Mount Cangshan in Dali, Yunnan, this study reached the following conclusions:

1. The PPO-BO algorithm consistently outperformed the DQN algorithm across all five plots and all six optimization schemes. Objective function values increased by up to 53.50% (plot P4), with an average improvement of 13.23% over DQN. The PPO-BO algorithm also converged approximately 35% faster, demonstrating superior computational efficiency for multi-objective stand structure optimization.
2. Incorporating slope (45° threshold) as a felling constraint improved, rather than diminished, optimization performance. Objective function values under the constrained scenario exceeded those of the unconstrained scenario across all plots, confirming that ecologically guided restriction of the candidate tree set enhances the quality of the policy network’s exploration.
3. The choice of tree selection strategy significantly affected optimization outcomes. The random selection scheme (M2) produced the highest average objective function value (0.4990), while the multiparameter evaluation scheme (M4) yielded the highest single-plot value (0.5255 in plot P5) and the largest relative advantage of PPO-BO over DQN (+7.73 percentage points). These results indicate that PPO-BO exhibits particularly strong compatibility with structured selection strategies.

Future work should extend the topographic constraint framework beyond slope to include aspect and soil type, integrate replanting with felling within a coordinated optimization framework, and incorporate individual-tree growth models to enable dynamic, multi-period stand management optimization.

## Data Availability

The raw data supporting the conclusions of this article will be made available by the authors, without undue reservation.
